# Exercise Attenuates Anabolic Steroids-Induced Anxiety via Hippocampal NPY and MC4 Receptor in Rats

**DOI:** 10.3389/fnins.2019.00172

**Published:** 2019-02-26

**Authors:** Jovana Joksimovic, Dragica Selakovic, Nemanja Jovicic, Slobodanka Mitrovic, Vladimir Mihailovic, Jelena Katanic, Dragan Milovanovic, Gvozden Rosic

**Affiliations:** ^1^Department of Physiology, Faculty of Medical Sciences, University of Kragujevac, Kragujevac, Serbia; ^2^Department of Histology and Embryology, Faculty of Medical Sciences, University of Kragujevac, Kragujevac, Serbia; ^3^Department of Pathology, Faculty of Medical Sciences, University of Kragujevac, Kragujevac, Serbia; ^4^Department of Chemistry, Faculty of Science, University of Kragujevac, Kragujevac, Serbia; ^5^Department of Pharmacology and Toxicology, Faculty of Medical Sciences, University of Kragujevac, Kragujevac, Serbia

**Keywords:** anabolic steroids, exercise, anxiety, neuropeptide Y, melanocortin 4 receptor, hippocampus

## Abstract

The aim of our study was to evaluate the effects of chronic administration of nandrolone-decanoate (ND) or testosterone-enanthate (TE) in supraphysiological doses and a prolonged swimming protocol, alone and in combination with ND or TE, on anxiety-like behavior in rats. We investigated the immunohistochemical alterations of the hippocampal neuropeptide Y (NPY) and melanocortin 4 receptor (MC4R) neurons, as a possible underlying mechanism in a modulation of anxiety-like behavior in rats. Both applied anabolic androgenic steroids (AASs) induced anxiogenic effect accompanied with decreased serum and hippocampal NPY. The exercise-induced anxiolytic effect was associated with increased hippocampal NPY expression. ND and TE increased the number of MC4R, while the swimming protocol was followed by the reduction of MC4R in the CA1 region of the hippocampus. However, NPY/MC4R ratio in hippocampus was lowered by AASs and elevated by exercise in all hippocampal regions. An augmentation of this ratio strongly and positively correlated to increased time in open arms of elevated plus maze, in the context that indicates anxiolytic effect. Our findings support the conclusion that alterations in both hippocampal NPY and MC4R expression are involved in anxiety level changes in rats, while their quantitative relationship (NPY/MC4R ratio) is even more valuable in the estimation of anxiety regulation than individual alterations for both NPY and MC4R expression in the hippocampus.

## Introduction

Although anabolic androgenic steroids (AASs) exert beneficial effects in therapeutically applied doses, this has been overshadowed by their abuse (Pope and Brower, 2004), with very serious behavioral consequences (Oberlander and Henderson, 2012). Beside various adverse effects, the neurotoxic effects following AASs misuse led to the plethora of behavioral disorders (Kanayama et al., 2008). Acute (Daly et al., 2003) and chronic (Wallin et al., 2015) treatments with high doses of AASs affect the neuroendocrine system exerting mood alterations. AASs abusers often perform exercise protocols in order to maintain lean body mass and muscular appearance (Davis and Scott-Robertson, 2000). Exercise protocols, in a manner of programmed aerobic physical exercise, showed beneficial effects on physical and mental health (Hillman et al., 2008). By modulating monoamine and serotonergic system, as well as influencing the opioid system, the physical activity in a form of programmed exercise training, represents a generally recommended regimen for preventing and treating various conditions in modern medicine (Anderson and Shivakumar, 2013). In addition, the alterations in hypothalamo-pituitary (HPA) axis, that are also confirmed to have a significant role in mood regulation, have been reported following AASs and exercise (Anderson and Shivakumar, 2013). However, the impact of those protocols on HPA axis markers in various tissues still remains inconsistent.

The hippocampus plays an important role in behavioral regulation. There are numerous evidence that administration of supraphysiological doses of AASs could modulate behavior through morphological and biochemical alterations in the hippocampus. Chronic administration of nandrolone-decanoate (ND) in supraphysiological doses exerted an anxiogenic effect by downregulation of gabaergic system in rat hippocampus (Selakovic et al., 2017). Moreover, the depressive-like behavior observed in rats following repeated ND treatments was accompanied by a reduction in the number of hippocampal neuropeptide Y (NPY) immunoreactive neurons, as previously described (Joksimovic et al., 2017a). As a structure with noticeable plasticity, the hippocampus can also be affected by exercise protocols, by means of increased cell proliferation and survival, with improved cognitive functions (van Praag et al., 2005; Erickson et al., 2011). A recent study concerning the behavioral effects of swimming training in rats showed that an antidepressant effect of exercise was accompanied by the increased number of hippocampal NPY interneurons (Joksimovic et al., 2017a).

A significant content of NPY was found in various brain regions involved in regulation of emotions, cognition, sleep and stress (Gehlert, 2004; Morales-Medina et al., 2010), and at the periphery (the sympathetic nervous system and adrenal medulla). The blood-brain barrier is permeable for NPY via a non-saturable transporter in rats, while that transporter has not been shown in humans (Kastin and Akerstrom, 1999). It has been noticed that serum levels of NPY were lower in patients with depression and anxiety symptoms (Ozsoy et al., 2016). Furthermore, the administration of NPY showed potential prophylactic (Serova et al., 2013) and curative beneficial effects on developed anxiety and depression (Serova et al., 2014). The reduced levels of NPY were detected in cerebrospinal fluid (CSF) of patients with major depression, reflecting disturbed synthesis, turnover or degradation of this peptide (Widerlov, 1988). Interestingly, the treatment with antidepressant drug restored NPY levels in CSF (Nikisch et al., 2005). Studies performed in rodents confirmed that anxiolytic effect was mediated by stimulation of Y1 and Y5 receptors (Sørensen et al., 2004) while the antidepressant action of NPY was achieved via Y1 receptors (Kotagale et al., 2013). Conversely, the results obtained in knockout mice showed the anxiogenic and prodepressant outcome of NPY action on Y2 and Y4 receptors (Painsipp et al., 2008). Since NPY receptors are localized on gabaergic neurons in stress-responsive brain regions, it seems reasonable that the interconnectivity between NPY and gabaergic system may underlie those behavioral alterations. Consequently, the anxiogenic action of single prolonged stress protocol in rats could also be considered as a result of a reduced number of immunoreactive NPY cells in the hippocampus after (Lee et al., 2018).

An alpha-melanocyte stimulating hormone (αMSH) also has a confirmed role in the modulation of anxiety and depression (Chaki and Okuyama, 2005). Five melanocortin receptors have been detected, but melanocortin receptor type 4 (MC4R) is predominantly expressed in the central nervous system, especially in brain regions responsible for stress responses and emotions control. Administration of αMSH, as well as its synthetic analog – melanotan II, resulted in a dose-dependent anxiogenic response in rats (Chaki et al., 2003). The anxiogenic effect observed following intracerebroventricular injection of αMSH in rats was attenuated by diazepam, suggesting that gabaergic system contributed to this behavioral effect of αMSH (Rao et al., 2003). On the other hand, intracerebroventricular administration of αMSH could reverse the anxiogenic effect induced by interleukin 1B via central MC4R (Cragnolini et al., 2005). The impact of central melanocortin system in the regulation of anxiety and depression levels was confirmed following intracerebroventricular administration of selective MC4R antagonist in rats (Kokare et al., 2010). In humans, peripheral αMSH (originating from the pituitary gland) levels correspond to the rate of diffusion from nucleus arcuatus (Forbes et al., 2001). Drastically decreased plasma αMSH levels were continuously detected in patients with anorexia nervosa (Galusca et al., 2015). Interestingly, increased levels of αMSH were detected in obese males, without an increase in adrenocorticoreleasing hormone (ACTH), suggesting that peripheral αMSH did not reflect central αMSH levels (Katsuki et al., 2000). Although derived from the same precursor, it seems that αMSH and ACTH secretions do not always occur in parallel. However, the relationship between central and peripheral αMSH has not been clarified yet.

The aim of this study was to evaluate the effects of administration of supraphysiological doses of two commonly abused AASs – ND or testosterone-enanthate (TE) alone or in combination with exercise protocol on anxiety level in rats, as well as the involvement of central and peripheral NPY and melatocortin system in the regulation of anxiety.

## Materials and Methods

### Ethics Statement

All research procedures were carried out in accordance with European Directive for the welfare of laboratory animals No. 86/609/EEC and principles of Good Laboratory Practice (GLP) and ARRIVE guidelines, approved by the Ethical Committee of the Faculty of Medical Sciences, University of Kragujevac, Serbia. Efforts were made to minimize the number of animals used and their suffering.

### Animals and Treatment

The total number of 48 male Wistar albino rats (3 months old, 350–400 g, obtained from the Military Medical Academy, Belgrade, Serbia) were housed in groups of four per cage. The rats were kept under controlled environmental conditions (23 ± 1°C, 12:12 h light/dark cycle – lights on 0800 h), with *ad libitum* access to food and water. Animals were divided into six groups (8 animals in each group): control group (C group), exercise group (E group), nandrolone-decanoate group (N group), exercise plus nandrolone-decanoate group (E+N group), testosterone-enanthate group (T group), and exercise plus testosterone-enanthate group (E+T group).

The rats from E group were submitted to swimming training (60 min per day, 5 consecutive days with 2 days break, for 6 weeks). Swimming was performed in groups of four animals, in heated (32 ± 1°C) glass tank (60 × 75 × 100 cm) with water depth of 60 cm. One week before starting the exercise protocols, the rats were familiarized with water contact by keeping them in a shallow tank with water (15 min/day) in order to reduce the water-induced stress (Contarteze et al., 2008). All rats in exercised groups were capable to swim for 60 min. The duration of single swimming trial was set within the range of swimming protocols that induced significant immunohistochemical changes in rat brain (Liu et al., 2010), and specifically in hippocampus (Joksimovic et al., 2017a; Selakovic et al., 2017), maintaining the characteristics of aerobic exercise (Araujo et al., 2015). The experimenter (blinded for the pretreatment protocols) was present in the room monitoring the rats during the entire swimming task. To avoid floating (that could seriously alter the level of exercise), as soon as it was observed, the experimenter pulled down the floating animal by the tail (the middle section) for 5–10 cm entirely in the water. The frequency of this manipulation gradually increased over time, starting from 2 to 3 per hour (at the beginning of the protocols) to 10–12 by the end of pretreatment protocols (probably due to increased adaptability of the animals). After completing the trial, towel drying was applied to the animals.

The applied doses of AASs (50–100 times higher compared to physiological androgen levels) were chosen to mimic human abuse (Kanayama et al., 2008). N group had received nandrolone-decanoate (20 mg/kg/weekly, s.c.) for 6 weeks (DEKA 300, SteroxLab, EU). T group was submitted to subcutaneous injections of testosterone-enanthate (Galenika, a.d., Serbia), at the same manner, by means of the same dose and treatment duration as N group.

The combined groups (E+N and E+T) underwent the same AASs protocols (as sedentary N and T group) while the exercise was simultaneously performed (the same swimming protocol as E group). The groups that have not received AASs, were submitted to the administration of approximately the equal volume of sterilized olive oil in the same manner as groups that received AASs. The rats from control and E groups were placed in water for 30 s each day of the training protocol, in order to eliminate the difference caused by immersion in water between the exercised and non-exercised groups.

Two days after completing the described protocols (no aggressive behavior was observed in the cages during pretreatment), the rats were placed in a testing room in order to acclimate (1–2 h) before the start of the behavioral testing. The animals were tested under proper conditions for this type of behavioral research (the noiseless room illuminated with controlled light, ∼100 lx). Behavioral testing was performed in the open field (OF) test, followed by an elevated plus maze (EPM) test, with the inter-trial interval of 15 min. The mazes were cleaned following each trial with water and ethanol (70%) in order to remove potentially interfering scents.

After completing the behavioral testing, the rats were anaesthetized by intraperitoneal application of ketamine (10 mg/kg) and xylazine (5 mg/kg), and sacrificed by decapitation. The brains were quickly removed for histological analysis, while the trunk blood samples were collected in frozen tubes, centrifuged (1700 × *g*, 10 min, 4°C) and serum/plasma samples were stored at -70°C for hormone analysis, as previously described (Milewicz et al., 2000).

### Behavioral Tests

#### OF Test

OF test is a commonly used behavioral test for the evaluation of general motor activity and anxiety levels in animal models. The apparatus consisted of a square arena (60 × 60 × 30 cm) made of black wood. The moving patterns were recorded by a digital video camera mounted centrally 150 cm above the aparatus. At the beginning of each trial, the rat was placed at the center of the arena, and the activity of rats was recorded for a period of 5 min. The following parameters were scored: cumulative duration in the center zone (CDCZ, with dimensions 30 × 30 cm), frequency in the center zone (FCZ), total distance moved (TDM, in cm), the percentage of time moving (%TM) during 5 min of testing, and the number of rearings. CDCZ and FCZ were considered as direct indicators of anxiety level. The time duration spent in the central part of OF arena was determined as the major index for anxiety, and more ambulation toward the central part of the open field reflected lower anxiety-like behavior. General locomotor activity was estimated by TDM and %TM, while the exploratory activity in the OF test was expressed by the number of rearings. Those parameters were considered as indirect markers of anxiety (decreased locomotion and exploration reflected high anxiety-like behavior).

#### EPM Test

EPM test was used to evaluate the anxiety level in rats. The maze consisted of two open (50 × 20 cm) and two enclosed arms (50 × 20 × 30 cm) and an open roof with the entire maze elevated 100 cm from the floor. Each rat was initially placed in the center of EPM (facing toward open arm) and free exploration was allowed. EPM test allows determining the emotional reactivity of animals by means of a conflict between secure parts (2 enclosed arms) and aversive parts of the maze (open arms). The activity of the rats was recorded for 5 min by a video camera mounted centrally 250 cm above the maze. The following parameters were recorded: cumulative duration in open arms (CDOA), frequency to open arms (FOA) – as direct indicators of anxiety level, while TDM (in cm) and %TM, as parameters of locomotion and the number of rearings, the number of head-dippings and the total exploratory activity (TEA) (Selakovic et al., 2017), as the parameters of exploratory activity, were considered as indirect indicators of anxiety (as described for OF test).

Video files were analyzed using Ethovision software [XT 12, Noldus Information Technology, the Netherlands].

#### Serum Hormone Assays

Serum samples were assessed for NPY and αMSH levels by Elecsys 2010 analyzer using the method of radioimmunoassay (RIA). Standard commercial kits (NPY RIA kit – RK-049-03, and αMSH RIA kit – RK-043-01, Phoenix Pharmaceuticals, Inc., Bunrlingame, United States) were used. NPY and αMSH levels were expressed in pg/ml and ng/ml, respectively. The sensitivity of the assays for NPY and αMSH were 69 pg/ml and 8.23 ng/ml, respectively. Inter- and intra-assay coefficients of variance were 12 and 5%, respectively, for NPY, and 15 and 7%, respectively, for αMSH, according to manufacturers’ specifications. Serum corticosterone concentrations were measured by immunoassay (R&D Systems Inc., Minneapolis, MN, United States), in duplicate within single assays, with an intra-assay CV of 8.0%. Plasma ACTH concentration was also determined without dilution, by a chemiluminescence method using an IMMULITE automatic analyzer (DPC, Los Angeles, CA, United States), in duplicate samples within a single assay, with an intra-assay CV of 9.6%.

#### Immunohistochemistry

Coronal brain FFPE sections, 5 μm thick, were incubated with rabbit polyclonal anti-NPY (1:250, AbD Serotec) overnight at room temperature. For NPY immunostaining we used Peroxidase Detection System (RE 7120-K, Novocastra, United Kingdom). For MC4 staining FFPE sections were incubated overnight with Anti-MC4 Receptor antibody (ab75506, Abcam, United Kingdom) diluted 1:250. For MC4 immunostaining EXPOSE Rabbit specific HRP/DAB detection IHC Kit (ab80437, Abcam, United Kingdom) was used according to the manufacturer’s protocol. For immunostaining we used rat cortex as a positive antibody control for NPY and MC4. Also, we performed a negative control with primary antibody omitted (secondary antibody only).

NPY and MC4R immunopositive neurons were counted using a light microscope (Carl Zeiss, Axioscop 40). The counting was always done on the dorsal hippocampus (3.80 mm caudal to the bregma, according to Paxinos and Watson stereotaxic atlas (Paxinos and Watson, 1998). A number of immunoreactive neurons was expressed per 1 mm^2^ for all regions (CA1, CA2/3, DG), using ImageJ software (NIH, United States).

Also, we counted the total number of immunoreactive neurons (NPY and MC4R) per (total) estimated hippocampal sections. Furthermore, we defined the new parameter – NPY/MC4R ratio that was calculated as a quantitative ratio between the total number of NPY and MC4R immunopositive neurons in two consecutive hippocampal sections. The counts were made by independent experimenters who were blind to the experimental protocol.

#### Statistical Analysis

The data were presented as means ± S.E.M. All obtained parameters were initially submitted to Levene’s test for homogeneity of variance and to Shapiro–Wilk test of normality. Comparisons between groups were performed using One-way ANOVA, followed by Tukey *post hoc* analysis. Simple linear regression analyses were performed to analyze relationships between parameters obtained in behavioral tests, serum hormone levels and histological data. Significance was determined at *p* < 0.05 for all tests, and for multiple comparisons at *p* ≤ 0.025. Statistical analysis was performed with SPSS version 20.0 statistical package (IBM SPSS Statistics 20).

## Results

### Behavioral Testing

**Figure 1:**
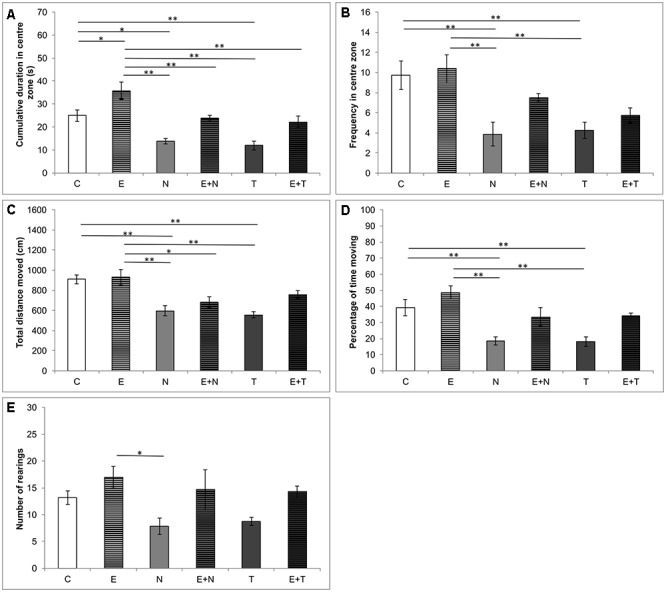
Parameters calculated from the open field test. **(A)** Cumulative duration in the center zone; **(B)** Frequency in the center zone; **(C)** Total distance moved; **(D)** Percentage of time moving; **(E)** Number of rearing; (C, control group; E, exercise group; N, nandrolone-decanoate group; E+N, exercise plus nandrolone-decanoate group; T, testosterone-enanthate group; E+T, exercise plus testosterone enanthate group). Bars represent means ± SEM, ^∗^ denotes a significant difference *p* ≤ 0.025, ^∗∗^ denotes a significant difference *p* ≤ 0.01.

The results obtained in OF test are presented in Figure 1. Both CDCZ (Figure 1A) and FCZ (Figure 1B) – direct indicators of anxiety level, were significantly altered by the applied protocols (*F* = 14.127 and *F* = 6.814, respectively, *df* = 5). The treatment with ND, as well as TE, significantly reduced CDCZ and FCZ compared to the control and exercise group (*p* < 0.01). On the other hand, the exercise protocol significantly increased CDCZ compared to the control group (*p* < 0.05). Moreover, the effect of swimming training in combined groups was sufficient to abolish the increase in anxiety-like behavior of the applied anabolics when compared to the control (Figure 1A,B). The applied protocols also induced significant changes in locomotor activity in OF test by means of TDM (Figure 1C, *F* = 8.988; *df* = 5) and %TM (Figure 1D, *F* = 8.969; *df* = 5). Both applied anabolics decreased TDM and %TM compared to the control (*p* < 0.01) and exercise (*p* < 0.01) groups. The number of rearings in OF test (Figure 1E) was significantly decreased (*p* < 0.05) in N group compared to E group (*F* = 3.307).

**Figure 2:**
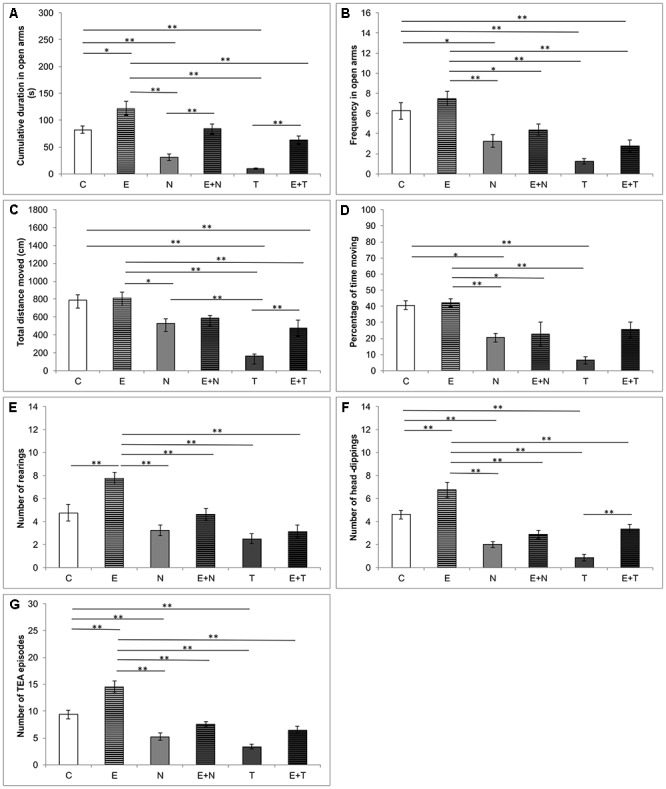
Parameters calculated from the elevated plus maze test. **(A)** Cumulative duration in open arms; **(B)** Frequency in open arms; **(C)** Total distance moved; **(D)** Percentage of time moving; **(E)** Number of rearings; **(F)** Number of head-dippings, **(G)** Number of TEA episodes; (C, control group; E, exercise group; N, nandrolone-decanoate group; E+N, exercise plus nandrolone-decanoate group; T, testosterone-enanthate group; E+T, exercise plus testosterone-enanthate group). Bars represent means ± SEM, ^∗^ denotes a significant difference *p* ≤ 0.025, ^∗∗^ denotes a significant difference *p* ≤ 0.01.

The anxiety levels in the EPM test, estimated by means of CDOA and FOA, were significantly affected by the treatments applied (*F* = 23.461 and *F* = 13.880, respectively, *df* = 5). As shown in Figure 2A,B, supraphysiological doses of ND and TE induced reduction of CDOA (*p* < 0.01) and FOA (*p* < 0.05 and *p* < 0.01, respectively), when compared to the control and exercise (*p* < 0.01) groups. However, the exercise protocol extended CDOA compared to the control group (*p* < 0.05), and observed lowering anxiety-like behavior of swimming training was also manifested by means of attenuation in AASs-induced decline in CDOA in combined groups when compared to the control group. Unlike in the combined E+N group, the exercise protocol was not sufficient to attenuate the increase of anxiety-like behavior of TE in combined E+T group, by means of FOA (*p* < 0.01). Parameters of locomotor activity in the EPM test, TDM and %TM, were also affected by protocols applied in this study (*F* = 16.321 and *F* = 10.153; *df* = 5, respectively). As shown in Figure 2C,D, ND (although not significant) and TE treatments resulted in decreased locomotion, compared to control, as well as to exercise group (*p* < 0.05 and *p* < 0.01, respectively). Although the exercise protocol did not significantly alter locomotor activity compared to the control, it was sufficient to increase the parameters of locomotion in the combined E+T (for TDM) compared to the sedentary T group (*p* < 0.01). It should be noticed that TDM in EPM test was significantly lower in the T group compared to the N group (*p* < 0.01). The strong negative effect of TE on locomotion was still prominent in the combined E+T group compared to the control (Figure 2C, *p* < 0.01). The analysis of the number of rearings (Figure 2E), the number of head-dippings (Figure 2F) and TEA episodes (Figure 2G), revealed significant alterations in exploratory activity in EPM test induced by applied protocols (*F* = 12.138, *F* = 25.151, and *F* = 30.222; *df* = 5, respectively). The exercise protocol resulted in the increase in exploratory activity by means of the number of rearings and TEA episodes (*p* < 0.01). On the other hand, the treatment with ND, as well as TE, significantly decreased the number of head-dippings and TEA episodes (*p* < 0.01) compared to the control group. The positive impact of exercise on the number of head-dippings was confirmed in the combined TE compared to the sedentary T group (*p* < 0.01).

### Serum Hormone Levels and Immunohistochemistry

**Figure 3:**
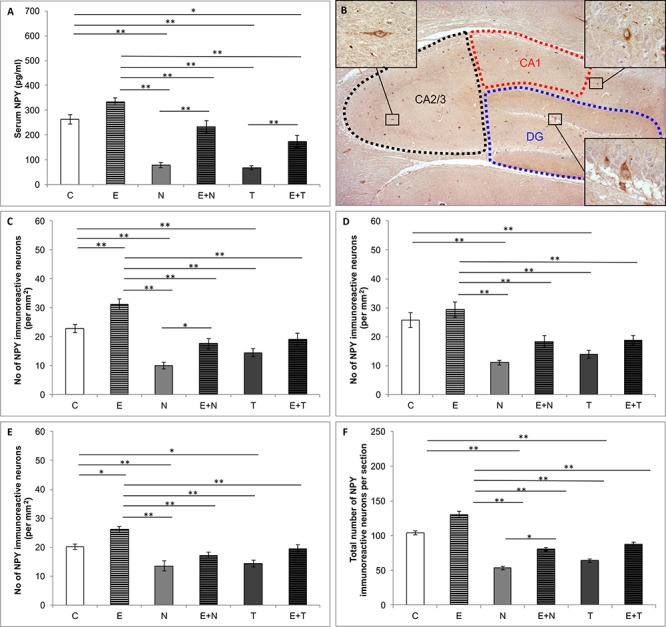
Quantification of NPY content. **(A)** Serum NPY levels; **(B)** Distribution of immunoreactive NPY neurons in hippocampus; **(C–F)** The number of NPY immunoreactive neurons in CA1 **(C)**, CA2/3 **(D)**, DG **(E)** and total hippocampal section **(F)**; (C, control group; E, exercise group; N, nandrolone-decanoate group; E+N, exercise plus nandrolone-decanoate group; T, testosterone-enanthate group; E+T, exercise plus testosterone-enanthate group). Bars represent means ± SEM, ^∗^ denotes a significant difference *p* ≤ 0.025, ^∗∗^ denotes a significant difference *p* ≤ 0.01.

Figure 3 depicts alterations in serum level of NPY (Figure 3A), distribution of NPY positive interneurons in the rat hippocampus (Figure 3B) and the number NPY immunoreactive neurons in CA1 (Figure 3C), CA2/3 (Figure 3D), DG (Figure 3E), and total hippocampal section (Figure 3F). Serum NPY levels were altered by the protocols applied (*F* = 34.120; *df* = 5). Both ND and TE treatment decreased levels of serum NPY (*p* < 0.01) compared to the control values. Although swimming training *per se* did not change serum NPY level when compared to the control group, it increased serum NPY levels in both combined groups compared to the sedentary N and T group (*p* < 0.01). However, unlike in the combined E+N group, serum NPY level in the combined E+T group was still below the control values (*p* < 0.05). The number of NPY immunoreactive neurons (per mm^2^) was significantly altered after chronic treatments applied in this study in all three estimated regions of hippocampus (CA1, *F* = 20.131; CA2/3, *F* = 12.762; DG, *F* = 13.457; *df* = 5), as well as in total number of NPY immunoreactive neurons per section (*F* = 21.871; *df* = 5). The exercise protocol produced an increase in the number of NPY positive interneurons in CA1 and DG of the hippocampus (*p* < 0.01 and *p* < 0.05, respectively), with no significant effect in CA2/3 region, as well as in total number of immunoreactive cells per section. Both applied AASs induced significant attenuation in a number of NPY positive interneurons in all estimated regions (*p* < 0.01in CA1 and CA2/3; *p* < 0.01 and 0.05 for ND and TE in DG, respectively) compared to the control group. The total number of NPY immunoreactive cells per section was also decreased by both applied anabolic steroids (*p* < 0.01). The exercise protocol alone produced a significant increase in the number of NPY positive interneurons per mm^2^ only in CA1 (*p* < 0.01), while the exercise-induced increase in NPY immunoreactivity in combined groups was manifested only for ND in CA1 region and total sections compared to the sedentary AASs groups (*p* < 0.05).

**Figure 4:**
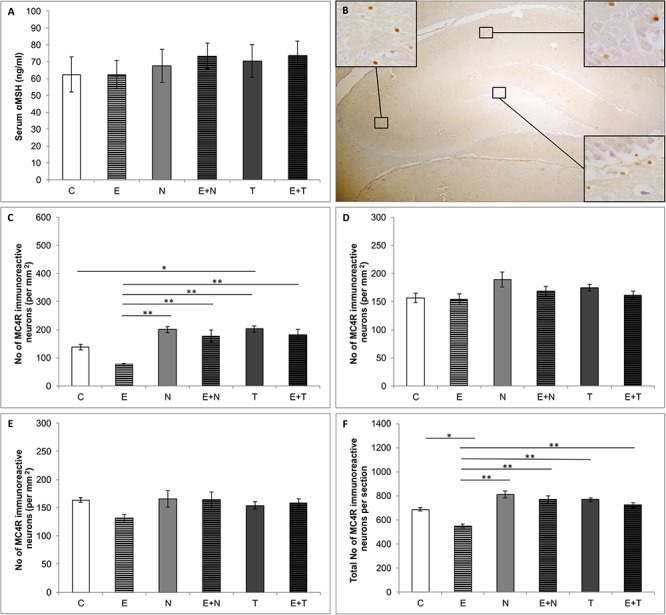
Serum αMSH and hippocampal MC4R content. **(A)** Serum αMSH levels; **(B)** Distribution of immunoreactive MC4R neurons in hippocampus; **(C–F)** Number of MC4R immunoreactive neurons in CA1 **(C)**, CA2/3 **(D)**, DG **(E)** and total hippocampal section **(F)**; (C, control group; E, exercise group; N, nandrolone-decanoate group; E+N, exercise plus nandrolone-decanoate group; T, testosterone-enanthate group; E+T, exercise plus testosterone-enanthate group). Bars represent means ± SEM, ^∗^ denotes a significant difference *p* ≤ 0.025, ^∗∗^ denotes a significant difference *p* ≤ 0.01.

Serum levels of αMSH (Figure 4A) were not altered by the applied protocols (*F* = 0.323, *df* = 5). Figure 4B shows the distribution of MC4R immunoreactivity in rat hippocampus in the control group. The protocols applied in this study significantly altered the number of MC4R positive cells in CA1 region (*F* = 12.368, *df* = 5), as well as the total number of MC4R immunoreactive cells per section (*F* = 10.718, *df* = 5), as shown in Figure 4C,F, respectively, while there were no alterations in CA2/3 (*F* = 2.095, *df* = 5, Figure 4D) and DG (*F* = 1.781, *df* = 5, Figure 4E) regions of the hippocampus. Although not significant in any individual hippocampal region, the effect of swimming training was manifested as mitigation of MC4R positive cells in total hippocampal section (*p* < 0.05). The chronic AASs administration did not significantly affect the number of MC4 immunoreactive neurons in total hippocampal sections, but TE administration significantly increased MC4R immunoreactivity in CA1 when compared to the control (*p* < 0.05). Total MC4R immunoreactivity per hippocampal section was decreased by exercise protocol (*p* < 0.05.

**Figure 5:**
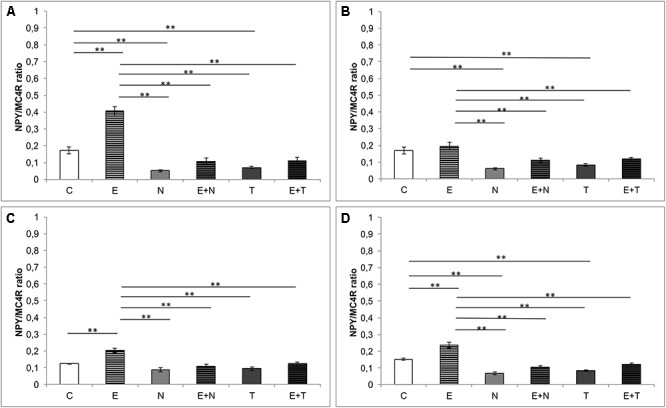
NPY/MC4 ratio in hippocampal regions. **(A)** CA1 region, **(B)** CA2/3 region, **(C)** DG, **(D)** per hippocampal section; (C, control group; E, exercise group; N, nandrolone-decanoate group; E+N, exercise plus nandrolone-decanoate group; T, testosterone-enanthate group; E+T, exercise plus testosterone-enanthate group). Bars represent means ± SEM, ^∗^ denotes a significant difference *p* ≤ 0.025, ^∗∗^ denotes significant difference *p* ≤ 0.01.

As shown in Figure 5, estimation of the new parameter – NPY/MC4R ratio, revealed significant alterations between groups in all three hippocampal regions (CA1, *F* = 73.576; CA2/3, *F* = 11.602; DG, *F* = 10.737, *df* = 5), as well as per total hippocampal sections (*F* = 34.390, *df* = 5). Both applied AASs induced a significant decrement of NPY/MC4R ratio compared to the control values in CA1, CA2/3 and total hippocampal section (*p* < 0.01). Exercise protocol resulted in strong augmentation of NPY/MC4R ratio in the CA1 and DG regions, as well as per total hippocampal section (*p* < 0.01). It should be noticed that the exercise protocol was not sufficient to reverse the decreased NPY/MC4R ratio in all investigated regions compared to the control values.

**Figure 6:**
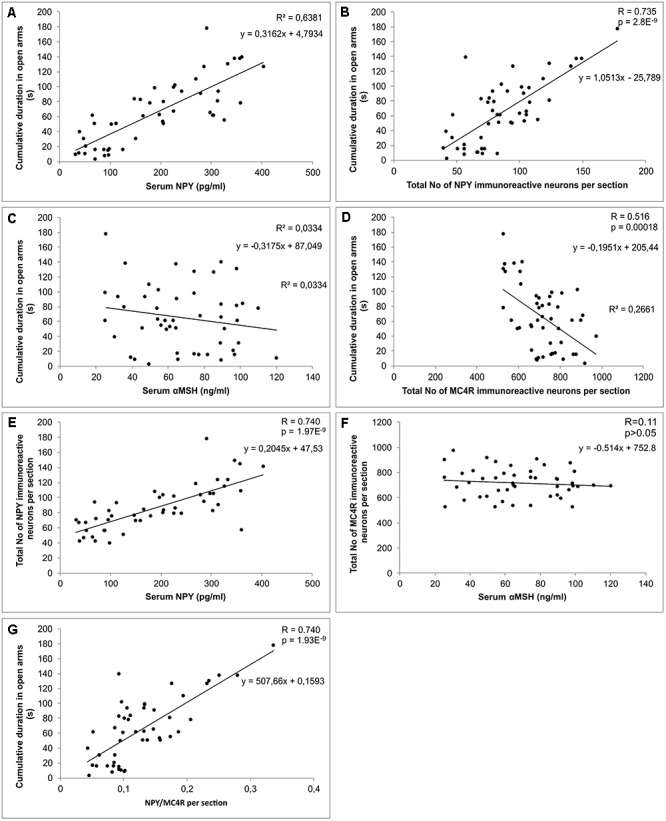
Relationships between biochemical, behavioral and immunohistochemical parameters. **(A)** serum NPY levels and cumulative duration in open arms, **(B)** total number of NPY immunoreactive neurons per hippocampal section and cumulative duration in open arms, **(C)** serum αMSH levels and cumulative duration in open arms, **(D)** total number of MC4R immunoreactive neurons per hippocampal section and cumulative duration in open arms, **(E)** serum NPY levels and total number of NPY immunoreactive neurons per hippocampal section, **(F)** serum αMSH levels and total number of MC4R immunoreactive neurons per hippocampal section, **(G)** hippocampal NPY/MC4R ratio and cumulative duration in open arms (*n* = 48).

Simple regression analysis showed a significant and strong positive correlation between CDOA and serum NPY levels (Figure 6A), while the serum levels of αMSH did not significantly correlate to CDOA (Figure 6C). Besides, CDOA significantly and positively correlated with the total number of NPY immunoreactive interneurons per section of the hippocampus (Figure 6B), while this behavioral parameter negatively correlated with the total number of MC4R positive cells per hippocampal section (Figure 6D). Analyzing the relationship between the total number of NPY immunopositive neurons per hippocampal section and serum NPY levels, it was observed (Figure 6E) that these two parameters significantly (positively) correlated. Nevertheless, the total number of MC4R immunopositive neurons per hippocampal section did not show a significant correlation with serum αMSH levels (Figure 6F). However, regression analysis revealed the strongest and the most significant correlation between CDOA and NPY/MC4R ratio (Figure 6G).

As shown in Table 1, none of the applied protocols significantly affected ACTH serum (*F* = 0.090) and corticosterone plasma (*F* = 0.102) levels.

**Table 1:** Blood levels of ACTH (plasma) and corticosterone (serum).

	C	E	N	E+N	T	E+T
ACTH (pg/ml)	21.66 ± 3.54	19.86 ± 3.94	23.05 ± 3.90	21.29 ± 4.87	20.55 ± 3.51	22.43 ± 3.74
Corticosterone (ng/ml)	99.31 ± 15.98	93.53 ± 11.73	99.93 ± 10.75	104.27 ± 10.29	99.95 ± 11.05	103.39 ± 10.38

*Values are expressed as mean ± SEM (n = 8). C, control group; E, exercise group; N, nandrolone-decanoate group; E+N, exercise plus nandrolone-decanoate group; T, testosterone-enanthate group; E+T, exercise plus testosterone-enanthate group.*

## Discussion

Epidemiological data implicate that AASs abuse represents a serious public health problem, exerting the behavioral manifestations accompanied with a variety of psychiatric disorders. The aim of this study was to evaluate the involvement of central and peripheral NPYergic and melanocortin system in modulation of alterations in anxiety levels following chronic AASs and exercise protocols. The results obtained in this study demonstrated that serum levels of NPY were significantly reduced by prolonged administration of supraphysiological doses of both applied AASs. This is in accordance with our previous report, concerning the chronic application of ND, where 6 weeks of ND administration (as performed in this study) induced 75% decrease of serum NPY levels (Joksimovic et al., 2017a). However, in this study, TE, as well as ND, induced a similar reduction in serum NPY levels compared to the control group (approximately 75 and 70%, respectively). Since there is no literature data describing the influence of TE supplementation on serum NPY levels, this result could only be interpreted as a similar effect produced by ND administration. Analyzing the behavioral effects of supraphysiological doses of both ND and TE, it was obvious that doses applied in this study produced a clear anxiogenic effect by means of anxiety indicators obtained in both OF and EPM test (Figure 1, 2). Beside the results presented in our recently published paper (Selakovic et al., 2017) for the anxiogenic effects of ND, the observed anxiogenic effect of ND and TE is also in line with literature data that showed the high potency of AASs (in supraphysiological doses) to induce anxiety-like behavioral manifestations in rats (Bueno et al., 2017), as well in humans (Lindqvist Bagge et al., 2017). Overall, it is clear that the AASs applied in this study produced the same type of alterations by means of various parameters. Indeed, certain quantitative differences between ND and TE actions were observed (significant for TDM in EPM, Figure 2C). Although AASs action strongly depends on the local synthesis and metabolism in hippocampus (Hojo et al., 2009), the exogenous administration may result in various behavioral responses probably due to different metabolic pathways. Namely, ND and TE belong to different classes and although both of them can be transformed into DHT (by the action of 5α-reductase), TE, like other testosterone esters can be aromatized into E2 (resulting in the opposite behavioral manifestations compared to testosterone), while ND can undergo this metabolic pathway with efficiency of only 20% compared to TE (Ryan, 1959; Winters, 1990). The main limitations of this preliminary study were that we did not explore specific underlying molecular mechanisms of the anxiety-like effects of AASs. However, based on the results of our previous study that showed increased oxidative damage in rat hippocapmal tissue following supraphysiological doses of AASs (manifested by increased ROS production and reduced antioxidant enzymes activity, Joksimović et al., 2017b), it seems possible that oxidative stress enhancement may be involved in consequent dysfunction of the neural circuit composed of several regions including the prefrontal cortex, amygdala, hypothalamus, anterior cingulated cortex, and other interconnected structures involved in emotions regulation (Kalinine et al., 2014).

In contrast to AASs effects, the prolonged exercise protocol, as a procedure that had a confirmed anxiolytic effect (Lalanza et al., 2015), resulted in elevated serum NPY levels (although without statistical significance). Since the exercise protocol applied in this investigation was performed in a manner of moderate intensity aerobic swimming training (Araujo et al., 2015), which apparently was not sufficient to induce significant elevation in serum NPY levels, our results do not correspond to previously reported increase in plasma NPY levels in rats following high intensity exercise (Keshtkar et al., 2014). A possible explanation could be found in different characteristics of exercise protocols. The moderate intensity (aerobic) protocol, as performed in our study, was not sufficient stressor that could lead to significant (probably compensatory) elevation of serum NPY levels that occurs simultaneously with the increase in catecholamine release (Lundberg et al., 1985). However, the exercise-induced elevation of serum NPY was significant in both combined compared to sedentary AASs groups. The increase in serum NPY in the combined groups was also accompanied with a clear decrease of anxiety-like manifestations compared to the sedentary AASs groups. The chronic exercise resulted in the reversal of the NPY levels to control values in E+N, but not in the E+T combined group. Interestingly, the stronger (negative) effect of TE on serum NPY was followed by the stronger anxiety-like effect of TE (Figure 2B,C) compared to ND in the combined groups. On the other hand, the decrease in anxiety-like behavior following exercise protocol was obvious by means of the most anxiety indicators obtained in OF and EPM tests. A similar effect of swimming training was also observed by Cruz et al. (2012). Although the protocols applied in this study (AASs and exercise) had opposite effects on the most behavioral manifestations of anxiety, as well as on the serum NPY levels, the existence of strong (positive) correlation between the serum levels of NPY and CDOA (the crucial behavioral parameter of anxiety), confirmed a clear interconnection of serum NPY and the anxiolytic response (Figure 6A). On the other hand, neither chronic AASs administration in supraphysiological dose nor prolonged exercise protocols (Table 1) was sufficient to significantly alter ACTH and corticosterone blood levels. Alsiö et al. (2009) demonstrated that supratherapeutic dose of ND (although lower than applied in this study) did not affect ACTH levels, but corticosterone plasma concentrations were significantly decreased. On the other hand, the effects of short-term administration of ND resulted in increased circulating levels of ACTH and corticosterone (Schlussman et al., 2000). In the same study where the brain regions responsible for mood regulation were estimated for early effects of ND on CRF mRNA levels, it was reported that mRNA levels in the amygdale were significantly reduced, and with no changes in hypothalamic CRF mRNA levels (Schlussman et al., 2000). The results of the study that investigated the effects of different AASs showed that trenbolone acetate induced significant reduction of corticosterone levels, while the effect of testosterone was opposite (Sillence and Rodway, 1990). Also, ACTH levels were not affected by the same protocol which is in accordance with the results obtained in this study. Interestingly, in gonadectomized rats the AASs supplementation reversed (lowered) the enhanced corticosterone levels in response to stress induction (Handa et al., 2013). The postulated mechanism for the way AASs affects HPA axis includes the alteration in 5α reductase activity in the brain tissue as a potential modulatory mechanism in the neuroendocrine stress response (Handa et al., 2013). Previous reports showed that prolonged swimming protocol did not affect levels of HPA axis serum markers, which is in line with the results obtained in the study, while brain corticosterone and CRF levels were augmented (Pietrelli et al., 2018). Furthermore, altered levels of corticosterone following long-term exercise significantly elevated plasma corticosterone levels in mice immediately after stress induction (Droste et al., 2003). In general, the diversity of reported alterations in HPA axis markers in literature following both types of protocols as performed in this study strongly suggest that final conclusion for the effect of AASs administration and exercise protocols crucially depends on specific characteristics of pretreatment protocols (class, dose, duration, etc. for AASs applied, and type, intensity, and duration of exercise), beside more complicated elements that involves age, sex, and species differences.

Beside the serum NPY levels, the protocols applied in this study also resulted in alterations of a number of hippocampal NPY immunoreactive cells. The NPY immunoreactivity in all three investigated regions of the hippocampus (CA1, CA2/3 and DG), as well as a total number of NPY cells per hippocampal section, was significantly reduced by both applied AASs. These findings are in accordance with our previous report concerning chronic ND administration (Joksimovic et al., 2017a). Moreover, literature data confirmed various alterations in NPY signaling produced by administration of other AASs in different brain regions. Chronic 17 α-methyltestosterone administration in adolescent rats resulted in eightfold decrease of NPY mRNA in VMN and threefold decrease of NPY mRNA in BNST, confirming the impact of AASs supplementation on central NPY system in regulation behavior (Ramos-Pratts et al., 2010). Moreover, ND also influenced hippocampal neurogenesis in DG cell culture by decreasing the number of newly born neurons (Brännvall et al., 2005). It has been reported that the increase in adult hippocampal neurogenesis could be responsible for reduced depression and anxiety behaviors in transgenic mice (Hill et al., 2015).

In contrast, the exercise protocol performed in our study resulted in augmentation of NPY immunoreactivity in investigated hippocampal regions (except in CA2/3), compared to control values, while the exercise-induced elevation in hippocampal NPY expression within the combined groups was only observed with ND (Figure 3C,D,F). This is in line with reported increased hippocampal NPY mRNA induced by swimming exercise protocol, accompanied with elevation of a brain-derived neurotrophic factor and VGF, which could synergistically influence mood alterations, by increasing synaptic plasticity and neurogenesis (Jiang et al., 2014). The relationship between NPY hippocampal immunoreactivity and anxiety behavior in our study was confirmed by strong (also positive) correlation between the total number of NPY per hippocampal section and CDOA (Figure 6B). Furthermore, the hippocampal NPY expression strongly correlated with the serum NPY levels (Figure 6E). Although previous reports did not confirm the linear relationship between plasma and CSF levels of NPY in human volunteers (Baker et al., 2013), our previous paper confirmed positive correlation between serum NPY levels and hippocampal immunoreactivity in rats (Joksimovic et al., 2017a). It is likely that the activity of local proteases in degradation of NPY could be responsible for the observed species differences. After all, it should be noted that the results for the NPY involvement in mood regulation presented in this preliminary study are only the first step in this complex subject. Further investigations should allow more specific insight in particular actions via different classes of NPY receptors in hippocampus. Even more, it will again require quantitative relationships among various classes of NPY receptors due to fact that the effects of stimulation of different receptors result in completely different behavioral outcomes (i.e., Y1R – anxiolytic and Y2R – anxiogenic effect, Wahlestedt et al., 1993; Thorsell et al., 2006).

In this study, none of the applied treatments induced alterations in serum αMSH levels (Figure 4A). Literature data demonstrated the increased plasma levels of αMSH only after high intensity exercise, but decreased in mildly exercised animals (Hiramoto et al., 2013). The observed alterations in plasma levels of αMSH were accompanied with changes in prohormone convertase 2 expressions. Therefore, it seems that the aerobic swimming training performed in our study was not of a sufficiently high intensity to induce a significant alteration in serum αMSH. To our knowledge, there is no literature data concerning the influence of AASs on serum αMSH levels.

Unlike for the NPY, the serum αMSH levels did not correlate with alterations in anxiety levels (Figure 6C). Interestingly, in patients with primary Sjogren’s syndrome (confirmed anxiety disorders with psychotic features), the positive correlation between anxiety (state and trait) with autoantibodies against αMSH in serum was reported (Karaiskos et al., 2010). Moreover, the increase in high-affinity autoantibodies against αMSH and decrease in serum levels of αMSH was accompanied with lower anxiety level in rats (Sinno et al., 2009). On the basis of those findings, the described mechanism of dysregulation in the melanocortin system seemed to be crucially involved in behavior control.

Quantification of the MC4R immunopositive neurons in hippocampus revealed that CA1 was the only region affected by performed protocols (Figure 4C), but the observed alterations in CA1were still sufficient to influence the total number of MC4R positive cells per section, as well (Figure 4F). The exercise protocols resulted in a reduction, while the AASs treatment induced the elevation of MC4R immunopositive neurons. These immunohistochemical alterations strongly and negatively correlated with behavioral manifestations of anxiety expressed by CDOA (Figure 6D). Our data are in accordance with the previous report which demonstrated that MC4R blockade led to anxiolytic effect (Serova et al., 2013). Moreover, the social isolation (as a stressogenic factor) resulted in decreased immunoreactivity of αMSH fibers in different brain regions involved in the regulation of anxiety and depression behavior (Kokare et al., 2010). Kokare and coworkers proposed an explanation that depleted αMSH immunoreactivity could be responsible for up-regulation MC4 receptors, which could lead to increased anxiety levels in social isolates. In line with that, the existence of a correlation between the total number of MC4R per hippocampal section and CDOA in our study could explain anxiety-like behavioral manifestations of administered AASs, as well as the elevation in number of MC4R positive cells.

As shown in Figure 6F, serum αMSH levels did not correlate with the number of MC4R immunopositive neurons in the hippocampus. Although numerous studies showed low or no permeability of blood-brain barrier for this peptide, it could not be definitively denied that systemic administration of αMSH had central effects trough melanocortin receptors distributed in various brain regions (Huang et al., 1998). Nevertheless, the absence of a linear correlation between peripheral αMSH levels and the number of hippocampal MC4R positive cells in our study suggests the complexity of this relationship.

Finally, since both the hippocampal NPY and melanocortin system, are involved in modulation of anxiety behavior in rats, we investigated the interconnection between those systems by means of NPY/MC4R expression ratio, as a new parameter that may be useful as a morphological background for the behavioral alterations. In all investigated hippocampal regions (Figure 5), NPY/MC4R ratio was significantly altered by the protocols applied. This ratio was elevated by swimming training in CA1 region (Figure 5A) and decreased by AASs protocols in CA1 and CA2/3 as well as in total hippocampal sections. The fact that the most significant (positive) correlation between NPY/MC4R ratio and the anxiety (Figure 6G) unequivocally confirms that this new parameter could be, due to its highest significance, a useful tool for estimation of anxiety level in behavioral investigations.

## Data Availability

All datasets generated for this study are included in the manuscript and/or the supplementary files.

## Author Contributions

JJ, DS, NJ, SM, VM, JK, DM, and GR designed and conducted the study, contributed to data collection, analyzed the data, reviewed the literature, and drafted the manuscript. All authors have read and approved the final version of the manuscript and agree with the order of presentation of the authors.

## Conflict of Interest Statement

The authors declare that the research was conducted in the absence of any commercial or financial relationships that could be construed as a potential conflict of interest.
